# Detecting Bulbar Involvement in Patients with Amyotrophic Lateral Sclerosis Based on Phonatory and Time-Frequency Features

**DOI:** 10.3390/s22031137

**Published:** 2022-02-02

**Authors:** Alberto Tena, Francesc Clarià, Francesc Solsona, Mònica Povedano

**Affiliations:** 1 CIMNE, Building C1, North Campus, UPC, Gran Capità, 08034 Barcelona, Spain; atena@cimne.upc.edu; 2 Department of Computer Science & INSPIRES, University of Lleida, Jaume II 69, 25001 Lleida, Spain; francisco.claria@udl.cat; 3 Neurology Department, Hospital Universitari de Bellvitge, L’Hospitalet de Llobregat, 08907 Barcelona, Spain; mpovedano@bellvitgehospital.cat

**Keywords:** ALS, bulbar involvement, voice, diagnosis, phonatory subsystem, time frequency, machine learning

## Abstract

The term “bulbar involvement” is employed in ALS to refer to deterioration of motor neurons within the corticobulbar area of the brainstem, which results in speech and swallowing dysfunctions. One of the primary symptoms is a deterioration of the voice. Early detection is crucial for improving the quality of life and lifespan of ALS patients suffering from bulbar involvement. The main objective, and the principal contribution, of this research, was to design a new methodology, based on the phonatory-subsystem and time-frequency characteristics for detecting bulbar involvement automatically. This study focused on providing a set of 50 phonatory-subsystem and time-frequency features to detect this deficiency in males and females through the utterance of the five Spanish vowels. Multivariant Analysis of Variance was then used to select the statistically significant features, and the most common supervised classifications models were analyzed. A set of statistically significant features was obtained for males and females to capture this dysfunction. To date, the accuracy obtained (98.01% for females and 96.10% for males employing a random forest) outperformed the models in the literature. Adding time-frequency features to more classical phonatory-subsystem features increases the prediction capabilities of the machine-learning models for detecting bulbar involvement. Studying men and women separately gives greater success. The proposed method can be deployed in any kind of recording device (i.e., smartphone).

## 1. Introduction

Amyotrophic lateral sclerosis (ALS) is a neurodegenerative disease with an irregular and asymmetric progression, characterized by a progressive loss of both upper and lower motor neurons and that leads to muscular atrophy, paralysis and death, mainly from respiratory failure. The life expectancy of patients with ALS is between 3 and 5 years from the onset of symptoms.

ALS causes muscle weakness and movement, speech, eating and respiratory impediments, leaving the patient reliant on caretakers and relatives and causing considerable social costs. Currently, there is no cure for ALS, although early detection can lead to the use of more appropriate therapies that may slow progress [1].

When the disease starts in the arms and legs, it is called spinal ALS (limb or spinal onset; 80% of cases), and when it starts in the cranial nerve nuclei, it is called bulbar ALS (bulbar onset; 20%). The bulbar muscle is responsible for speech and swallowing, so patients with the later variant have a shorter life expectancy. However, dysarthria, or slurred or difficult speech articulation, affects 80% of all ALS patients [2]. In bulbar ALS, these symptoms usually appear at the onset of the disease, while in spinal ALS, they appear later. Early detection of bulbar involvement in those with ALS is crucial for better diagnosis and prognosis, and could be the key to effectively slowing the development of the disease.

The authors in [3,4,5] demonstrated that the deterioration of the bulbar muscle affected some phonatory-subsystem features. Among these were jitter, shimmer, harmonic-to-noise ratio (HNR), pitch, formant trajectories, correlations of formants with articulation patterns, fractal jitter, and Mel Frequency Cepstral Coefficients. Consequently, as suggested in previous works [6,7,8,9,10,11,12,13], imperceptible changes in speech and voice can be detected through objective measures.

Time-frequency representation (TFR), broadly applied to detecting several conditions [14,15,16,17,18], has been recently used to detect pathological changes in voice signals [19]. TFR enables the evolution of the periodicity and frequency components to be observed over time, allowing the analysis of non-stationary signals, such as voice signals [20]. The spectrogram is the most common TFR for the analysis of audio signals. This representation corresponds to Cohen’s class of time-frequency energy distributions in general. The depiction of a spectrogram is not optimal in terms of resolution quality. There are Cohen class representations that have greater resolution quality. They are all made by smoothing the Wigner distribution, which has the finest resolution but the most detrimental interference. The smoothing functions chosen strike a balance between resolution quality and the elimination of detrimental interference terms.

The authors in [21] used TFR representations from the Cohen class for the onset signal of the vocal fold to diagnose various phonation problems induced by pathological alterations. To assess the voice signal, Cohen class TFRs were combined with a cone kernel distribution to provide optimum smoothness across time. The authors demonstrated that even minor pathogenic alterations in the vocal folds can be seen in TFR, allowing for sensitive affection detection and diagnosis.

In ALS, voice and speech impairment can occur up to 3 years before a diagnosis [22], and when the bulbar muscle function is damaged, voice and speech deteriorates significantly as the disease advances [23]. Features obtained from Cohen class TFRs could aid in the identification of bulbar involvement even earlier than human hearing can.

Centering attention on the subject at hand, R. Norel et al. [24] developed machine-learning models that recognize the presence and severity of ALS based on a variety of frequency, spectral, and voice quality characteristics. An et al. [25] employed Convolutional Neural Networks to classify the intelligible speech of ALS patients compared to healthy people. Finally, Gutz et al. [26] combined SVM and feature filtering techniques.

Based on previous works, and starting from our recent studies [6,18], our paper suggests using phonatory-subsystem [6] and time-frequency [18] features jointly. This also is our hypothesis and main contribution. These features, extracted from a portion of the five Spanish vowels, could enhance the performance of the classification models for the early detection of bulbar involvement, for which the main goals (and contributions) of this research are:

1. To design a new methodology for the automatic detection of bulbar involvement in males and females based on phonatory-subsystem and time-frequency features.
2. To obtain a set of statistically significant features for diagnosing bulbar involvement efficiently.
3. To analyze the performance of the most common supervised classification models to improve the diagnosis of bulbar involvement.

## 2. Methods

### 2.1. Participants

Of the 65 participants selected for this study, 14 of those with ALS had been diagnosed with bulbar involvement (11 females and 3 males; mean = 56.8 years, standard deviation = 12.3 years), 33 had ALS but had not been diagnosed with bulbar involvement (8 females and 25 males; mean = 57.6 years, standard deviation = 12.0 years) and 18 were healthy individuals (9 females and 9 males; mean = 45.2 years, standard deviation = 12.2 years). The main clinical records of the ALS participants are summarized in Table 1. It can be seen that the sample is well age-balanced.

The ALS patients’ voices were checked by a multidisciplinary clinical team and finally selected by a neurologist for this study.

The control subjects were recruited through personal advertisements in the hospital facilities by the researchers involved in this study. After contacting the volunteers, they received an information sheet explaining the procedure and goal of the study as well as the exclusion criteria. They were interviewed through a questionnaire and those who did not report any voice issue or relevant previous condition were selected for the study.

The control subjects were recruited through personal advertisements conducted in the hospital facilities by the researchers involved in this study. Most of them were companions of ALS patients. After contacting them, control subjects received an information sheet explaining the procedures and goals of the study as well as the exclusion criteria. Control subjects were informed that the study focused on voice analysis to distinguish bulbar involvement in ALS patients. They were interviewed through a questionnaire. Those who did not report any voice issue or relevant previous condition were selected for the study. When they were eligible and still willing to participate, they were invited to come to the hospital room where the voice samples were registered.

### 2.2. Vowel Recording

There are five vowel segments in the Spanish phonological system (a, e, i, o, u). These were obtained and analyzed from each ALS patient, all of whom were Spanish speakers.

Under medium vocal loudness conditions, each participant uttered a sustained sample of each Spanish vowel for 3–4 s. The recordings were made in a standard hospital room using a laptop and a USB EMITA Streaming GXT 252 microphone calibrated for dBSPL. It has a sensitivity of −35 dBSPL and a maximum sound pressure level of 135 dBSPL. The participants sat on a chair with the microphone positioned approximately 30 centimeters from their mouths. The voice signals were recorded using *Audacity*, an open-source application [27], at a sampling rate of 44.100 Hz and 32-bit quantization.

A visual inspection of the spectrograms of the voice signals was conducted similarly to the procedure in [28] to analyze the signal type of the participants’ voices. Their results suggested four voice types, of which only type 1 and type 2 were considered suitable for perturbation analysis.

In this study, all the control subjects presented type 1 voice signals, which were periodic without strong modulations or subharmonics. They showed multiple clearly and nearly straight defined harmonics.

Among the 14 ALS patients with bulbar involvement, 10 patients presented type 1 voice signals, which were nearly periodic and showed some clearly defined harmonics. However, a small amount of noise was observed in some voices (four of them). Four of the ALS patients with bulbar involvement presented type 2 voice signals. These had some strong modulations and subharmonics, yet still presented stable and periodic segments in their voices.

Among the 33 ALS patients without bulbar involvement, 29 presented type 1 voice signals, which were nearly periodic and showed multiple or at least some clearly defined harmonics. Instead, four of them presented type 2 voice signals with some strong modulations and subharmonics but still with stable and periodic segments.

It was observed that most of the information of the signal recordings was contained in the range from 0 to 4000 Hz. Therefore, it was decided to decimate all the recording signals sampled at 44.100 Hz using a decimated factor of 5. Signals re-sampled at 8820 Hz were obtained.

Then, each re-sampled signal was standardized by means of the z-score technique. The z-score measures the distance of a signal sample from the mean of the re-sampled signal in terms of the standard deviation. The resulting standardized signal had mean 0 and standard deviation 1, and retained the shape properties of the re-sampled signal. For the re-sampled signal with mean $\bar{X}$ and standard deviation *S*, the z-score of a signal sample *x* was computed as:

(1) $$z=\frac{\left(x-\bar{X}\right)}{S}$$

Finally, a segment of 150 ms of each re-sampled and standardized signal (x(t)) was chosen for analysis by tacking the midpoint at the center of the phonation.

### 2.3. Phonatory-Subsystem Features

A total of 15 features from the phonatory subsystem defined in [6,13] were used. They were computed by means of the standard methods used in Praat [29] and the setting details used were the same as in [6]. These features were:

- Fundamental period cycle-to-cycle variation (**Jitter(absolute)**, Equation (2)).
  (2) $$Jitter\left(absolute\right)=\frac{1}{N-1}\sum_{i=1}^{N-1}\left|T_{i}-T_{i-1}\right|,$$
  where *N* is the number of cycles and $T_{i}$ the duration of the *i*th cycle.
- Relative period (**Jitter(relative)**, Equation (3)).
  (3) $$Jitter\left(relative\right)=\frac{\frac{1}{N-1}\sum_{i=1}^{N-1}\left|T_{i}-T_{i-1}\right|}{\frac{1}{N}\sum_{i=1}^{N}T_{i}}\times 100$$
- Relative perturbation (**Jitter(rap)**, Equation (4)).
  (4) $$Jitter\left(rap\right)=\frac{\frac{1}{N-1}\sum_{i=1}^{N-1}\left|T_{i}-\frac{1}{3}\sum_{n=i-1}^{i+1}T_{n}\right|}{\frac{1}{N}\sum_{i=1}^{N}T_{i}}\times 100$$
- Five-point period perturbation quotient (**Jitter(ppq5)**, Equation (5)).
  (5) $$Jitter\left(ppq5\right)=\frac{\frac{1}{N-1}\sum_{i=2}^{N-2}\left|T_{i}-\frac{1}{5}\sum_{n=i-2}^{i+2}T_{n}\right|}{\frac{1}{N}\sum_{i=1}^{N}T_{i}}\times 100$$
- Variability of the peak-to-peak amplitude (**Shimmer(dB)**, Equation (6)).
  (6) $$Shimmer\left(dB\right)=\frac{1}{N-1}\sum_{i=1}^{N-1}|20\times log\left|\left(\frac{A_{i+1}}{A_{i}}\right)\right|,$$
  where $A_{i}$ is the extracted peak-to-peak amplitude data and *N* is the number of extracted fundamental periods.
- Relative amplitudes of consecutive periods (**Shimmer(relative)**, Equation (7)).
  (7) $$Shimmer\left(relative\right)=\frac{\frac{1}{N-1}\sum_{i=1}^{N-1}\left|A_{i}-A_{i+1}\right|}{\frac{1}{N}\sum_{i=1}^{N}A_{i}}\times 100$$
- Three-, five- and eleven-point amplitude perturbation (**Shimmer(apqP)**, Equation (8)).
  (8) $$Shimmer\left(apqP\right)=\frac{\frac{1}{N-1}\sum_{i=1}^{N-1}\left|A_{i}-\left(\frac{1}{P}\sum_{n=i-1}^{i+1}A_{n}\right)\right|}{\frac{1}{N}\sum_{i=1}^{N}A_{i}}\times 100,$$
  where P = {3, 5 and 11}.
- Mean and standard deviation (**HNR(mean)** and **HNR(SD)**) of the harmonics-to-noise-ratio (HNR, Equation (9)).
  (9) $$HNR=10\times log_{10}\frac{r(T_{0})}{1-r(T_{0})},$$
  where $r(T_{0})$ is the second local maximum of the normalized auto-correlation function and $T_{0}$ is the period of the signal.
- Mean, standard deviation, minimum and maximum value of the pitch (**pitch(mean)**, **pitch(SD)**, **pitch(min)** and **pitch(max)**). See [29] for more details about obtaining the pitch.

### 2.4. Time-Frequency Features

The methods employed to obtain the time-frequency features were inspired by the previous work, presented in [16,17], and implemented with MATLAB [30].

First, the Wigner distribution (WD) of the real signal x(t) of each voice segment was obtained and convoluted with the Choi-Williams exponential function. The resulting Choi-Williams distribution was normalized ($CWD_{N}\left(f,t\right)$). For more details, see [18].

Then, the joint probability density distribution pD(f,t) (Equation (10)) was obtained.

(10) $$pD\left(f,t\right)=m_{t}\left(t\right)\cdot m_{f}\left(f\right),$$

where $m_{t}\left(t\right)$, instantaneous power, and $m_{f}\left(f\right)$, spectral energy density, are the marginal density functions of $CWD_{N}\left(f,t\right)$.

According to Equation (10), pD can be only computed as the product of the marginal density functions $m_{t}\left(t\right)$ and $m_{f}\left(f\right)$ (of $CWD_{N}$) if they are statistically independent. To corroborate this assumption, we computed the joint time-frequency moments of the $CWD_{N}$ ($\langle t^{n}f^{m}\rangle$ from n=1 and m=1 to n=15 and m=15 where *n* and *m* are the frequency and time moment orders) of the vowels of all the participants. All of these were 0 or very close to 0. This confirmed the statistical independence of $m_{t}\left(t\right)$ and $m_{f}\left(f\right)$.

pD(f,t) is completely free of interference and negative values. Thus, it is very useful for extracting time-frequency features for classification.

**Figure 1:**
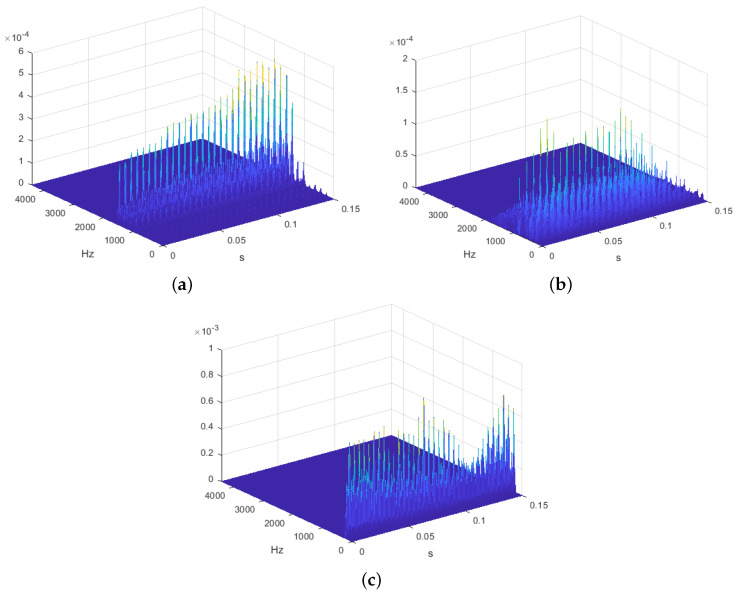
pD(f,t) of vowel “*a*” for 3 different patients with bulbar involvement. The marked difference in the graphic representation of the time-frequency between the subjects can be clearly appreciated. (**a**) Patient pD without bulbar involvement. (**b**) Patient pD with slight bulbar involvement. (**c**) Patient pD with severe bulbar involvement.

Figure 1 shows the comparison of the pD(f,t) of the vowel “*a*” from three different patients. Non-undesirable effects were observed in the pD(f,t). Figure 1a corresponds to a patient without bulbar involvement. The pD(f,t) shows a voice rich in harmonics. Figure 1b shows the pD(f,t) of the vowel “a” of a patient diagnosed with slight bulbar involvement. Significant differences can be observed. Voice harmonics appear attenuated. Figure 1c shows the pD(f,t) of an even more extreme case, diagnosed with severe bulbar involvement. It can be seen that its voice harmonics appear even more attenuated.The visual appraisal of these figures clearly shows the significant differences in the pD(f,t) between ALS patients with and without bulbar involvement.

From the pD(f,t), a set of 30 features per vowel was obtained. Twenty-one features were computed by dividing the spectrum (0–4410 Hz) into 7 frequency bands. These were 1, 0–80 Hz; 2, 80–250 Hz; 3, 250–550 Hz; 4, 550–900 Hz; 5, 900–1500 Hz; 6, 1500–3000 Hz; 7, 3000–4410 Hz. These bands were selected to capture the differences observed in the time-frequency representations of the two groups of ALS patients by means of the visual appraisal of pD(f,t) in the range of these frequency bands. These features were:

- Average instantaneous spectral energy (E(t), Equation (11)) for each frequency band (**E_Bn1**…**E_Bn7**).
  (11) $$E\left(t\right)=\int_{f_{1}}^{f_{2}}pD\left(f,t\right)df,$$
  where $f_{1}$ and $f_{2}$ are the lower and upper frequencies of each band.
- Instantaneous frequency peak (f_Cres(t), Equation (12)) for each frequency band (**f_Cres1** …**f_Cres7**).
  (12) $$f\_Cres\left(t\right)=\frac{1}{E(t)}argmax_{f}\left[\prod_{f_{1}}^{f_{2}}f\cdot pD\left(f,t\right)\right]$$
- Average instantaneous frequency ($f_{mi}\left(t\right)$, Equation (13)) of the spectrum for each frequency band (**f_Med1**…**f_Med7**).
  (13) $$f_{mi}\left(t\right)=\int_{f_{1}}^{f_{2}}\frac{1}{E(t)}f\cdot pD\left(f,t\right)df$$

10 additional features were added:

- Instantaneous (**H_t**, Equation (14)) and spectral (**H_f**, Equation (15)) information entropies. Furthermore, the joint Shannon entropy (**H_tf**, Equation (16)) was also used.
  (14) $$H\_t=-\int log_{2}\left(m_{tN}\left(t\right)\right)\cdot m_{tN}\left(t\right)dt,$$
  where $m_{tN}\left(t\right)$ is the quantified instantaneous marginal obtained from the $m_{t}\left(t\right)$ and $m_{fN}\left(f\right)$ is the quantified frequency marginal obtained from the $m_{f}\left(f\right)$.
  (15) $$H\_f=-\int log_{2}\left(m_{fN}\left(f\right)\right)\cdot m_{fN}\left(f\right)df$$
  (16) H_tf=H_t+H_f
- Spectral information (IE(f), Equation (17)), for each frequency band (**IE_Bn1**…**IE_Bn7**).
  (17) $$IE\left(f\right)=-log_{2}\left(m_{fN}\left(f\right)\right)$$
- Kurtosis (**K**, Equation (18)).
  (18) $$K=\langle m_{t}{\left(t\right)}^{n}m_{f}{\left(f\right)}^{m}\rangle ,$$
  where n=4 and m=0.
- Joint time-frequency moment ($\langle t^{n}f^{m}\rangle$,^18^,^31^]) where *n* and *m* (n, m = 1, 7, 15) are the frequency and time moment orders, i.e., the following time-frequency moments were used: $\langle t^{1}f^{1}\rangle$, $\langle t^{7}f^{7}\rangle$ and $\langle t^{15}f^{15}\rangle$.

### 2.5. Feature Selection

From a total of 65 participants, 18 were labelled C (healthy group), 14 were labelled B (ALS patients with bulbar involvement) and 33 were labelled NB (ALS patients without bulbar involvement). Furthermore, every ALS participant was labelled A.

Accordingly, four classification problems were analyzed, males and females being studied separately, these being C vs. B, C vs. NB, B vs. NB and C vs. A.

The Multivariant Analysis of Variance (MANOVA), which uses the covariance between the features in testing the statistical significance of the mean differences, was performed in IBM SPSS Statistics [32] to select a subset of relevant features for use in constructing the classification model for these four cases. This procedure made it possible to contrast the null hypothesis in the features obtained.

To perform this statistical analysis, it was assumed that the features had a multivariable normal distribution, and no assumptions were made regarding the homogeneity of the variance or the correlation between the features. A significance value of *p*-value < 0.05 was considered sufficient to assume the existence of feature differences between the four groups analyzed.

### 2.6. Classification Models

Several supervised classification models were implemented in R [33] to measure the classification performance. These models were fitted with the features selected. These were standardized by subtracting the mean and centered at 0. Ten-fold cross-validation was implemented in R using the caret package to draw suitable conclusions. This consisted of dividing the dataset into 10 contiguous chunks, each containing approximately the same number of samples, and then performing 10 training-testing experiments as follows: for each chunk i∈{1,2,…,10}, the current chunk was retained for testing the model and training was performed on the remaining 9 chunks, recording the results. The average performance of the 10 training-testing experiments was finally provided.

The upsampling technique with replacement was applied to the training data by making the group distributions equal to deal with the unbalanced dataset, which could bias the classification models [34].

The supervised models with classification thresholds of 50% were built in R [33]. In binary classification problems, the classification threshold is a value that converts the model prediction to positive or negative depending on whether the prediction is above or below the threshold.

The classification algorithms used were the most popular ones in ALS: Support Vector Machine (SVM), Neural Networks (NN), Linear Discriminant Analysis (LDA), Logistic Regression (LR) and Random Forest (RF). For more details, see [6].

### 2.7. Model Validation Metrics

There are various metrics for evaluating classification models [35]. The foremost among these, accuracy, sensitivity and specificity, were used to evaluate the performance of the classification models.

## 3. Results

First, the significant features from the four cases (C vs. B, C vs. NB, B vs. NB and C vs. A) were selected. Then, the performance of the classification models was evaluated.

### 3.1. Selecting the Significant Features

From the 50 features obtained, the MANOVA analysis was applied to select those that were statistically significant. Four comparisons were analyzed separately for males and females: C vs. B, C vs. NB, B vs. NB and C vs. A. Features not showing statistical significance (*p*-value ≥ 0.05) were discarded.

Table 2 shows the significant features obtained for males. In the C vs. B case, this was a set of 12 statistically (half phonatory) significant features (*p*-value < 0.05); in C vs. NB, there were 13 (10 of them phonatory); in B vs. NB, 9 (all time-frequency); and in C vs. A, 12 (10 of which were phonatory).

For females (Table 3), in the C vs. B case, a set of 20 statistically significant features (*p*-value < 0.05) was obtained (13 out of 20 were phonatory). In the C vs. NB case, a set of 10 statistically significant features was obtained (6 of them, phonatory). In the case B vs. NB, a set of 14 statistically significant features was obtained (12 of which were phonatory). In the C vs. A case, 20 statistically significant features were obtained (12 being phonatory).

### 3.2. Classification Models

The classification models were fitted with the significant features selected in Section 3.1. Table 4 and Table 5 show the classification performance for males and females, respectively. The results are presented for the *accuracy*, *sensitivity* and *specificity* of the models used for the four cases.

For males in C vs. B case, all the classifiers generally performed well. RF obtained the best *accuracy*, 96.1%. For LDA and NN, *accuracy* was 95.0% and for SVM and LR, 93.3% and 91.9% respectively. LR gave the best *sensitivity* (95.0%), and RF and LDA the best specificity=97.5%.

Similar performance was achieved in C vs. NB and C vs. A cases. In these, SVM was the best model (an *accuracy* of 93.1% was reached for C vs. NB and 92.6% for C vs. A).

Otherwise, the outcomes worsened in B vs. NB compared with the other cases. Despite RF obtained the best *accuracy* (91.8%), the *sensitivity* it achieved was the worst.

For females, in the C vs. B case, the results also indicate that the performance of all classifiers was excellent. RF gave the best *accuracy*, 98.1%, *sensitivity*, 96.6%, and *specificity*, 100%.

Similar behavior was obtained in the C vs. NB and C vs. A cases. In these, RF was also the best model (obtaining *accuracy* of 94.1% and 95.8% for C vs. NB and C vs. A respectively). In both cases, LDA achieved the best *specificity*.

Meanwhile, the results were worse in B vs. NB compared with the other cases. Although RF obtained the best *accuracy* at 84.8%, the outcomes obtained with it for *specificity* and especially *sensitivity* were very low.

In general, the best model was RF. Special attention should be paid to female outcomes. Poor results were obtained for both genders in the B vs. NB case.

## 4. Discussion

### 4.1. Principal Findings

The results obtained demonstrate that it is possible to diagnose bulbar involvement using supervised gender-specific models fitted to the significant phonatory and time-frequency features.

**Table 2:** Significant Features for males.

Comparison	Feature	*p*-Value
C vs. B	*shimmer(dB)*	0.039
	*shimmer(apq11)*	<0.001
	*pitch(mean)*	0.001
	*pitch(SD)*	0.023
	*pitch(min)*	0.016
	*pitch(max)*	<0.001
	*f_Cres2*	0.046
	*f_Cres6*	0.046
	*f_Med2*	<0.001
	*f_Med6*	0.008
	*K*	0.027
	$\langle t^{1}f^{1}\rangle$	0.002
C vs. NB	*jitter(relative)*	0.008
	*shimmer(dB)*	0.001
	*shimmer(relative)*	0.008
	*shimmer(apq3)*	0.035
	*shimmer(apq11)*	<0.001
	*pitch(mean)*	0.001
	*pitch(SD)*	0.002
	*pitch(min)*	0.023
	*pitch(max)*	0.001
	*HNR(mean)*	0.037
	*IE_Bn1*	0.045
	*H_tf*	0.015
	*H_f*	0.045
B vs. NB	*f_Cres1*	0.044
	*f_Cres2*	0.028
	*f_Med2*	<0.001
	*f_Med6*	0.011
	*f_Med7*	0.024
	*H_tf*	0.009
	*H_f*	0.009
	*K*	0.045
	$\langle t^{1}f^{1}\rangle$	<0.001
C vs. A	*jitter(relative)*	0.009
	*shimmer(dB)*	0.001
	*shimmer(relative)*	0.009
	*shimmer(apq3)*	0.044
	*shimmer(apq11)*	<0.001
	*pitch(mean)*	0.001
	*pitch(SD)*	0.002
	*pitch(min)*	0.015
	*pitch(max)*	<0.001
	*HNR(mean)*	0.046
	*H_tf*	0.048
	$\langle t^{1}f^{1}\rangle$	0.034

**Table 3:** Significant Features for females.

Comparison	Feature	*p*-Value
C vs. B	*jitter(relative)*	0.001
	*jitter(absolute)*	<0.001
	*jitter(rap)*	<0.001
	*jitter(ppq5)*	<0.001
	*shimmer(relative)*	<0.001
	*shimmer(dB)*	<0.001
	*shimmer(apq3)*	<0.001
	*shimmer(apq5)*	<0.001
	*shimmer(apq11)*	<0.001
	*pitch(mean)*	<0.001
	*pitch(SD)*	<0.001
	*pitch(max)*	<0.001
	*HNR(mean)*	<0.001
	*f_Cres2*	0.004
	*f_Cres6*	0.029
	*f_Cres7*	0.020
	*E_Bn2*	0.003
	*f_Med2*	<0.001
	*f_Med6*	0.013
	$\langle t^{1}f^{1}\rangle$	0.028
C vs. NB	*jitter(absolute)*	<0.001
	*shimmer(apq11)*	<0.001
	*pitch(mean)*	<0.001
	*pitch(SD)*	0.003
	*pitch(min)*	0.008
	*pitch(max)*	<0.001
	*f_Cres7*	0.011
	*E_Bn2*	0.015
	*f_Med1*	0.014
	$\langle t^{7}f^{7}\rangle$	0.022
B vs. NB	*jitter(relative)*	<0.001
	*jitter(absolute)*	<0.001
	*jitter(rap)*	<0.001
	*jitter(ppq5)*	<0.001
	*shimmer(relative)*	<0.001
	*shimmer(dB)*	<0.001
	*shimmer(apq3)*	<0.001
	*shimmer(apq5)*	<0.001
	*shimmer(apq11)*	<0.001
	*pitch(SD)*	<0.001
	*pitch(max)*	0.029
	*HNR(mean)*	<0.001
	*H_tf*	0.026
	*H_f*	0.048
C vs. A	*jitter(relative)*	<0.001
	*jitter(rap)*	0.001
	*jitter(ppq5)*	0.004
	*shimmer(relative)*	<0.001
	*shimmer(dB)*	<0.001
	*shimmer(apq3)*	<0.001
	*shimmer(apq5)*	0.001
	*shimmer(apq11)*	<0.001
	*pitch(mean)*	<0.001
	*pitch(SD)*	<0.001
	*pitch(max)*	<0.001
	*HNR(mean)*	0.003
	*f_Cres2*	0.006
	*f_Cres7*	0.005
	*E_Bn2*	0.003
	*f_Med1*	0.049
	*f_Med2*	0.001
	*f_Med7*	0.049
	*H_t*	0.039
	$\langle t^{1}f^{1}\rangle$	0.018

In the case of B vs. C, the accuracy achieved was up to 98.1% (RF) and 96.1% (RF) for females and males, respectively.

Lower performance was obtained in C vs. NB but this was still higher than expected. The voice performance in C or NB should be similar. This indicates that some participants in the NB group were probably incorrectly diagnosed. This is coherent with [6]. Similarly, the excellent performance achieved in C vs. A suggests that some of the members of A (14 out of 47) have bulbar involvement. Alternatively, although the most stable segments of the voice samples were selected for analysis, many co-articulatory effects could have influenced the results. Moreover, phonatory-subsystem features are subject to inherently large variability, even for Cs.

On the whole, huge uncertainty was observed in the evaluation concerning bulbar involvement among the participants in the NB group. The case of B vs. NB disclosed that the models did not differentiate between the B and NB subject groups as well as they did with the other groups. RF achieved the best overall performance (accuracy = 91.8%) in males. However, the model presented problems for spotting positive cases (sensitivity = 55.0%). In females, RF achieved an *accuracy* of 84.8%. These values are still far from the ones obtained in the C vs. B case. These outcomes additionally reinforce the idea that NB subjects were misdiagnosed.

The outcomes of each comparison between groups depend on the significant features chosen (between phonatory and time-frequency). In other words, the optimal results in each experiment are obtained with an ad-hoc set of features. This means the differentiation between the participants in different groups depends on different features. However, classifiers obtained very similar results for each experiment, showing a lesser influence.

The results obtained proved that combining phonatory-subsystem and time-frequency features improves the ability of the machine-learning models to detect bulbar involvement. In addition, detecting bulbar involvement also depends on the ad-hoc set of significant features found for such a case.

**Table 4:** Performance of male models. RF: Random Forest; LR: Logistic Regression; LDA: Linear Discriminant Analysis; NN: Neuronal Networks; SVM: Support Vector Machines.

		C vs. B	C vs. NB	B vs. NB	C vs. A
RF	Accuracy	**96.1**	91.9	**91.8**	92.0
	Sensitivity	86.1	**92.1**	55.0	**93.8**
	Specificity	**97.5**	91.0	**97.5**	87.0
LR	Accuracy	91.9	89.2	88.5	91.3
	Sensitivity	**95.0**	90.3	75.0	90.7
	Specificity	92.0	86.9	89.5	94.0
LDA	Accuracy	95.0	91.1	81.3	92.0
	Sensitivity	85.0	88.6	**90.0**	90.7
	Specificity	**97.5**	98.0	80.5	96.0
NN	Accuracy	95.0	90.0	86.1	92.0
	Sensitivity	90.0	91.3	75.0	91.5
	Specificity	95.0	86.5	88.4	93.0
SVM	Accuracy	93.3	**93.1**	86.1	**92.6**
	Sensitivity	85.0	91.2	85.0	90.7
	Specificity	95.0	**98.0**	86.7	**98.0**

### 4.2. Comparison with Prior Work

This study is consistent with [6,7,8,36] which demonstrated that such phonatory-subsystem features as jitter, shimmer, pitch and HNR were sensitive indicators for describing pathological voices in ALS. It is also consistent with [6] where great uncertainty was found in the diagnosis of NBs participants.

**Table 5:** Performance of female models. RF: Random Forest; LR: Logistic Regression; LDA: Linear Discriminant Analysis; NN: Neuronal Networks; SVM: Support Vector Machines.

		C vs. B	C vs. NB	B vs. NB	C vs. A
RF	Accuracy	**98.1**	**94.1**	**84.8**	**95.8**
	Sensitivity	**96.6**	**92.5**	**92.3**	**95.8**
	Specificity	**100**	95.5	**75.0**	96.0
LR	Accuracy	91.4	93.0	74.7	91.3
	Sensitivity	91.3	90.0	75.0	93.4
	Specificity	91.5	95.5	**75.0**	87.0
LDA	Accuracy	93.1	90.4	72.1	90.7
	Sensitivity	87.6	82.5	70.0	87.3
	Specificity	86.6	**97.5**	**75.0**	**98.0**
NN	Accuracy	93.2	86.9	71.1	90.6
	Sensitivity	93.3	85.0	72.3	93.6
	Specificity	94.0	89.0	70.0	84.5
SVM	Accuracy	95.1	91.6	74.2	93.6
	Sensitivity	93.3	90.0	73.6	94.7
	Specificity	97.5	93.0	**75.0**	91.5

Besides the 15 phonatory-subsystem features obtained in [6], this study also provides 35 time-frequency features. The combination of phonatory-subsystem and time-frequency features, after performing MANOVA for feature selection, enhanced the outcomes of [6], which achieved the best results to date for detecting bulbar involvement in ALS using only acoustic features, ahead of [8,13,24].

*Accuracies* of up to 98.1% (RF) and 96.1% (RF) for females and males respectively were achieved when comparing the bulbar and control participants (case B vs. C). This *accuracy* exceeded the one obtained in [24] with SVM (79.0%) by 17.1% for males and 15.1% for females. The other studies found did not distinguish the classification problems by gender. In [6], SVM obtained an accuracy of 95.8%. In [13], NN based on Mel Frequency Cepstral Coefficients (coefficients for speech representation based on human auditory perception) obtained 90.7%. In [8], NN based on phonatory-subsystem features obtained 91.7% and adding motion sensors for both lip and tongue data increased the accuracy to 96.5% at the expense of including more invasive measurements. For females, our results outperformed those from the aforementioned studies by 2.3%, 7.4% and 6.4% respectively. For males, ours were 0.3% above those obtained in [6] and 5.4% and 4.4% above those obtained in [8,13].

When comparing ALS patients diagnosed with bulbar involvement with those patients in whom bulbar involvement has yet to be detected (B vs. NB), the outcomes outperformed the ones obtained in [6]. The respective accuracy for males and females increased by 16.3% and 9.3% with the same classifier (RF) (91.8% and 84.8% as against 75.5%). This is an important outcome which indicates that the use of time-frequency features increases the identification of bulbar involvement among patients with ALS.

The outcomes obtained in the C vs. NB and C vs. A cases were very similar to those in [6], reinforcing the idea that some NBs could have bulbar involvement.

The most important gains were obtained when comparing B and NB. The selection of the significant features for this comparison improved the outcomes. Thus, involvements (i.e., bulbar) could be detected through a separate, and more closely adjusted, set of features. Consequently, by increasing the identification of particular features, treatment could be better customized for each ALS patient.

In addition, only studies showing C vs. B have been presented in the literature (except in [6]). No attempts to distinguish other subjects have been made to date. We highlight this differentiating issue, and the importance of future research into it.

### 4.3. Limitations

The use of classification models with small datasets hinders the full assessment of the importance of the findings. The size of the dataset was, in part, determined by the low prevalence of ALS, which is considered a rare disease. The small number of samples in the B group was influenced by the heterogeneity of the ALS disease in which patients’ symptomatology is very diverse.

Furthermore, hand editing the segments of the voice recordings is inherently subjective and may introduce subtle and unintended selection biases. Although automatic instruments have been created, these methods are currently insufficiently accurate and require manual correction.

## 5. Conclusions and Future Work

This research directly addresses a recent statement released by the NEALS bulbar subcommittee regarding the need for methodologies based on objective measurements [37]. The outcomes achieved reinforce the idea that machine learning can be a suitable tool for helping with the diagnosis of ALS with bulbar involvement using common recording or mobile (i.e., smartphone) devices.

We demonstrate the usefulness of assessing bulbar involvement properly using phonatory-subsystem and time-frequency features from a study of the Spanish vowels that outperformed previous works, specifically [6,8,13,24]. It was also demonstrated that group identification depends on the significant features found for such an experiment.

The main contribution is the differentiation of diagnosis by gender. This outperformed all the results in the literature.

The next steps of this work will consist of improving the corpus for diagnosing bulbar dysfunction. It is planned to increase the sample size and enhance the annotation of the ALS patients without bulbar involvement. Novel methods based on the creation of vowel patterns and semi-supervised classification models will be developed to provide hints for distinguishing those ALS patients without bulbar involvement who may have been misdiagnosed.

Vowel patterns could be generated from the quasi-periodic components of a short stable segment of the five Spanish vowels. Principal and independent component analysis of these patterns is also envisioned.

Moreover, additional research is required to develop this concept properly. Longitudinal research studies are conceived in which patients’ diagnoses are obtained at multiple follow-ups. Several repetitions of the sustained phonations will be required to minimize sampling variability even for the control subjects.

## Figures and Tables

**Table 1:** ALS participants clinical records. Notation: Age (in years). ALSFR-R (Rating Scale-Revised): scores (0–48) the severity of ALS; Bulbar: Bulbar involvement; NA: Data not available.

Age	Sex	ALSFR-R	Bulbar	Bulbar Onset Symptoms
37	F	37	NO	No Symptoms
38	M	6	YES	NA
39	M	43	NO	No Symptoms
41	M	34	NO	No Symptoms
41	M	34	NO	No Symptoms
43	F	21	YES	Dysphagia
44	F	19	NO	No Symptoms
48	F	36	NO	No Symptoms
48	F	29	YES	Dysphagia
48	M	31	NO	No Symptoms
48	M	45	NO	No Symptoms
49	M	NA	NO	No Symptoms
50	M	39	NO	No Symptoms
52	M	43	NO	No Symptoms
52	F	27	YES	Dysphagia
52	M	33	NO	No Symptoms
53	F	29	YES	Dysphagia/Dysarthria
55	M	26	NO	No Symptoms
55	M	24	NO	No Symptoms
56	M	35	NO	No Symptoms
56	M	27	NO	No Symptoms
58	F	46	YES	Dysarthria
58	M	28	YES	NA
59	F	33	YES	NA
60	M	46	YES	NA
63	M	22	NO	No Symptoms
63	M	42	NO	No Symptoms
63	M	NA	NO	No Symptoms
65	M	24	NO	No Symptoms
66	F	41	NO	No Symptoms
67	M	NA	NO	No Symptoms
67	F	33	YES	Dyspnoea
68	M	NA	NO	No Symptoms
68	F	21	NO	No Symptoms
69	M	37	NO	No Symptoms
70	F	28	YES	Dysphagia
70	F	17	NO	No Symptoms
70	M	46	NO	No Symptoms
70	M	27	NO	No Symptoms
70	F	23	YES	Dysphagia/Dysarthria
71	M	39	NO	No Symptoms
71	F	32	YES	Dysphagia
72	M	30	NO	No Symptoms
72	F	38	NO	No Symptoms
76	F	30	NO	No Symptoms
81	M	36	NO	No Symptoms
81	M	28	NO	No Symptoms
84	F	30	YES	NA

## Sample Availability

Source and Synthetic Data Repository. All the models implemented in R are available in a GitHub repository in *ELA source files* (https://github.com/atenad/ALS), accessed on 27 January 2022. A synthetic dataset of the features obtained is also provided. It was built in R from the original values using the synthpop package [38].

## Abbreviations

The following abbreviations are used in this manuscript:

TFR	Time-frequency representation
Jitter (absolute)	inter-cycle variation of the fundamental period
Jitter(relative)	relative period
Jitter(rap)	relative perturbation
Jitter(ppq5)	five-point period perturbation quotient
Shimmer(dB)	Variability of the peak-to-peak amplitude
Shimmer(relative)	relative amplitudes of consecutive periods
Shimmer(apqP)	three, five and eleven-point amplitude perturbation
HNR	harmonics-to-noise ratio
HNR(mean)	mean HNR
HNR(SD)	standard deviation of HNR
WD	Wigner distribution
CWD	Choi-Williams exponential function
pD	joint probability density distribution
E(t)	average instantaneous spectral energy
$f_{mi}\left(t\right)$	average instantaneous frequency
f_Cres(t)	instantaneous frequency peak
H_t	instantaneous information entropy
H_f	spectral information entropy
H_tf	joint Shannon entropy
IE(f)	spectral information
K	kurtosis
$\langle t^{n}f^{m}\rangle$	joint time-frequency moment
SVM	Support Vector Machine
NN	Neural Networks
LDA	Linear Discriminant Analysis
LR	Linear Logistic Regression
RF	Random Forest
C	control group
B	group of ALS participants diagnosed with bulbar involvement
NB	group of ALS participants not diagnosed with bulbar involvement
A	group of ALS participants with or without bulbar involvement
